# Regulatory Effects of SLC7A2‐CPB2 on Lymphangiogenesis: A New Approach to Suppress Lymphatic Metastasis in HNSCC

**DOI:** 10.1002/cam4.70273

**Published:** 2024-10-09

**Authors:** Kai Song, Yanshi Li, Kai Yang, Tao Lu, Min Wang, Zhihai Wang, Chuan Liu, Ming Yu, Mengna Wang, Zhaobo Cheng, Min Pan, Guohua Hu

**Affiliations:** ^1^ Department of Otorhinolaryngology The First Affiliated Hospital of Chongqing Medical University Chongqing China; ^2^ Department of Otorhinolaryngology The Affiliated Hospital of Guizhou Medical University Guiyang China

**Keywords:** CPB2, head and neck squamous cell carcinoma, lymph node metastasis, lymphangiogenesis, *SLC7A2*

## Abstract

**Background:**

Lymph node metastasis (LNM) is a critical factor affecting the outcomes of head and neck squamous cell carcinoma (HNSCC) and the main reason for treatment failure. This study was designed to examine the effects of the key genes involved in the LNM of HNSCC.

**Methods:**

Tissue samples (HNSCC) were examined by transcriptome sequencing, and the core genes associated with LNM were detected via bioinformatics analysis. The functions of these core genes were then validated using the TCGA biological database and their effects on the propagation, invasion, and metastasis of HNSCC cells were evaluated through cell culture experiments. Moreover, the effect of core gene expression on the LNM capability of HNSCC was confirmed via a footpad xenograft mice model.

**Results:**

In the findings, a key gene involved in the LNM of HNSCC was identified as *SLC7A2*. It was correlated with adverse clinical prognosis and expressed with low expression in HNSCC tissues. As shown in cell culture experiments, FaDu and SCC15 cell growth, invasion, and migration were inhibited when *SLC7A2* was overexpressed. Further, cell apoptosis was stimulated, and lymphangiogenesis was suppressed through the downregulation of CPB2 expression. Animal studies demonstrated that the growth and LNM of HNSCC cells were inhibited by *SLC7A2* overexpression.

**Conclusion:**

It is concluded that *SLC7A2* is involved in HNSCC lymphatic metastasis by controlling CPB2 function. The results are anticipated to offer new directions for the effective treatment of HNSCC.

## 1 Introduction

Head and neck squamous cell carcinoma (HNSCC) is a prevalent cancer that arises in the buccal cavity, larynx, hypopharynx, nasopharynx, and nasal cavity. It is responsible for 543,000 fatalities yearly and has an estimated annual incidence of 100 million cases worldwide [1]. Despite the progress observed with treatments, the overall survival (OS) for HNSCC patients has not shown significant improvement recently [2, 3, 4]. Particularly for patients with advanced HNSCC, the survival rate remains low at 30 to 40% due to high tumor infiltration and lymph node metastasis (LNM) [5, 6, 7]. Consequently, it is crucial to identify precise prognostic markers as well as understand the mechanisms underlying the progression of LNM.

The prognosis for HNSCC is considerably affected by LNM and tumor infiltration [8, 9]. In previous studies, abnormal gene expression has been marked as a possible factor in LNM formation. For instance, the VEGFC/VEGFR3 and PROX1 signaling axis is widely recognized as the primary driver of lymphangiogenesis in many solid tumors [10, 11]. Lymphangiogenesis can also be regulated by the expression of neuropilin‐2 (NRP2) and SEMA3F; the SEMA3F‐NRP2 axis functions as a biomarker to predict a possibility of occult LNM [12]. Furthermore, the ANXA6/TRPV2 axis has been shown to promote LNM in HNSCC by stimulating autophagy [13]. Nevertheless, the exact mechanisms underlying this process are still unclear, and patients have not benefited substantially from these advances. Thus, it is essential to examine the mechanism of LNM in HNSCC to establish an effective treatment strategy.

Genomic features have the potential to serve as prognostic markers and therapeutic targets [14, 15]. As a member of the solute carrier superfamily, *SLC7A2* encodes CAT2, a transmembrane transporter that facilitates the absorption and transportation of amino acids. Recent research demonstrated a correlation between reduced *SLC7A2* expression and an unfavorable prognosis in patients with ovarian cancer and hepatocellular carcinoma (HCC) [16, 17]. Furthermore, mice lacking *SLC7A2* exhibit high levels of tumor‐promoting M2 macrophages and an increased risk of inflammation‐related colon tumorigenesis due to the mediation of various chemokines [18]. Nevertheless, the function of *SLC7A2* in HNSCC is still unknown and necessitates further study.

Carboxypeptidase B2, also known as human thrombin‐activated fibrinolysis inhibitor (TAFI), transforms a fibrinolytic reaction when stimulated by plasmin, thrombin, or the thrombin/thrombomodulin (TM) complex [19]. It increases considerably in breast cancer tissues and exhibits a strong association with lymphovascular infiltration and interleukin 10 (IL‐10) expression. It has been demonstrated that siRNA suppression of TAFI inhibits breast cancer cell migration and invasion [20], suggesting that CPB2 might have a crucial role in lymphangiogenesis. Conversely, the precise mechanism by which it functions remains unclear.

Besides, LNM acts as a crucial prognostic risk factor for HNSCC, exerting an influence on the OS of the patients. In the present study, differential and core genes related to LNM of HNSCC were successfully detected via whole‐transcriptome sequencing and bioinformatics analysis. This study highlighted new perspectives on the molecular mechanism by which core genes regulate LNM in HNSCC. Further, the study provides possible ways to identify the potent targets for HNSCC treatments.

## 2 Materials and Methods

### 2.1 Collection of HNSCC Tissues

There were 79 HNSCC patients enrolled in the study who underwent surgical procedures at the First Affiliated Hospital of Chongqing Medical University, China from 2016 to 2018. This study comprised the following inclusion criteria: Patients were initially diagnosed with HNSCC without any concurrent malignant tumor complications; preoperative imaging techniques (CT scan, ultrasound, or MRI) were used to assess the extent of lesions; there were no indications of distant and hemorrhagic metastasis. No previous chemotherapy, radiotherapy, or targeted therapy was administered. The sample was collected as per the guidance of the Ethical Committee of the institute and the Declaration of Helsinki. Patients submitted informed consent forms before participation, and the study was designed under the guidance of the above‐mentioned ethical committee.

### 2.2 Microarray Analyses

The library was constructed and sequenced by China Science and Technology Genomics Co., Ltd. (Shanghai, China). Whole‐transcriptomic sequencing was performed on 10 fresh‐frozen HNSCC samples, comprising five cases with LNM and five cases without. Raw reads were filtered using fastp software (https://github.com/OpenGene/fastp). Sequence alignment on the clean reads was conducted with Hisat2 (https://github.com/trinityrnaseq/trinityrnaseq/wiki). We utilized the Stringtie software to count the fragments within each gene region following alignment. The reference genome version is Human Genome Reference Consortium (GRCh38) (ftp://ftp.ensembl.org/pub/release‐91/fasta/homo_sapiens/dna/Homo_sapiens.GRCh38.dna.toplevel.fa.gz). Subsequently, we normalized the counts using the trimmed mean of M values (TMM) algorithm (http://www.kegg.jp/), and ultimately calculated the Fragments Per Kilobase Million (FPKM) values for each gene. In the calculation of FPKM values, the ‘K’ stands for kilobase, which is used to normalize the impact of gene length. As for the gene annotation file, I use the GTF format (GCF_000001405.38_GRCh38.p12_genomic.gtf) because it is widely supported and used in gene expression analysis. Differential analysis of gene expression between the two groups was performed using the edgeR software package. After calculating the *p*‐value, multiple hypothesis testing corrections were applied to obtain the *Q* value. Additionally, the differential expression fold, referred to as fold change (FC), was calculated based on the FPKM value, often expressed as log2 (FC). The criteria for screening differentially expressed genes in this project were set at *Q* value 2.

### 2.3 RNA Isolation and qPCR Analysis

Fresh‐frozen samples were processed for RNA extraction using an E.Z.N.A. Total RNA Kit I (Omega Bio‐tek, USA). The isolated RNA was used as a template for cDNA synthesis at 37°C for 15 min followed by 70°C for 5 min via a PrimeScript RT Reagent Kit (Takara, China). The qPCR experiment was conducted using the SYBR PrimeScript RT‐PCR Kit (Takara). The housekeeping control (reference gene) was *GAPDH*, and the corresponding gene expressions were estimated via the 2^−ΔΔ^Ct method.

The primer sequences were as described below:

- *GAPDH* (sense): CAGCGACACCCACTCCTC
- *GAPDH* (antisense): TGAGGTCCACCACCCTGT
- *SLC7A2* (sense): TTTCCCAATGCCTCGTGTAATC
- *SLC7A2* (antisense): TGCACCCGATGACAAAGTAGC
- *CPB2* (sense): ATGCGAACAATCATTGCATCG
- *CPB2* (antisense): CAGTAGGTTTCCGAGCATGAG
- *VEGFC* (sense): GAGGTCAAGGCTTTTGAAGGC
- *VEGFC* (antisense): CTGTCCTGGTATTGAGGGTGG
- *PROX1* (sense): AGAAGGGTTGACATTGGAGTGA
- *PROX1* (antisense): TGCGTGTTGCACCACAGAATA

### 2.4 Protein Analysis via Western Blotting (WB)

The whole‐cell lysate assay kit (KeyGEN BioTECH, China) was used to extract total protein from tumor cell lines and tissues by the established protocol. The experiment of WB was conducted as per the defined procedure. Primary antibodies targeting SLC7A2 (1:1000), CPB2 (1:1000), VEGFC (1:1000), PROX1 (1:1000), and GAPDH (1:2000) were obtained from Abcam (ab13379, ab9546, ab199359, and ab8245) and Thermo Fisher (PA5‐89910). Goat anti‐rabbit IgG (Beyotime, China) secondary antibodies were also used with 1.5000 dilutions.

### 2.5 Immunohistochemistry (IHC)

Tumor tissue sections (4‐μm) were embedded in paraffin and stained with IHC reagent (SP‐9000, China). Tissue sections (on sides) were processed for dewaxing and rehydrated in sequential grades of xylene and alcohol solutions. Antigen retrieval was carried out for 15 min at 95°C using citrate buffer. This step was followed by 15 min of incubation at 37°C with an endogenous peroxidase blocker. The section slides were kept with primary antibodies; SLC7A2 (1:200, Thermo Fisher), PA5‐89910, AND LYVE1 (1:200, Abcam, ab33682) at 4°C for 24 h. Subsequently, the sections were placed for 15 min with HRP‐linked streptavidin, after which they were counterstained with hematoxylin and visualized with diaminobenzidine (DAB). Three random fields were selected for the detection of each section using a light microscope at 200× magnification. Further, the scores of signaling intensity and the scores of staining distribution were multiplied to calculate the final scores. The scores of signal intensity were divided into different categories: without signal (0), weak (1), moderate (2), and strong (3). Based on the proportion of positive cells, the staining distribution scores were as follows: 0 (0%–5%), 1 (5%–25%), 2 (25%–50%), 3 (50%–75%), and 4 (75%–100%).

### 2.6 Cell Culture

Cells (FaDu and SCC15) were procured from the Cell Bank of the Chinese Academy of Sciences (Shanghai, China). The cells were allowed to grow under the standard culture condition in Dulbecco's Modified Eagle's Medium (DMEM; Gibco, USA) enriched with 1% antibiotics (penicillin/streptomycin) (Beyotime, China) and 10% fetal bovine serum (FBS; Gibco, Australia).

### 2.7 RNA Interference and Cell Transfection

The siRNA was purchased from GenePharma (Shanghai, China). Cells (2.5 × 10^5^ cells/well) were seeded in culture plates and grown until they reached 60%–70% confluency. They were transfected with siRNA via GP‐Transfect‐Mate and incubated for 6 h in FBS‐free DMEM. The exhausted medium was then changed with 10% FBS‐enriched DMEM and the cells were maintained further for 48 to 72 h. Total RNA and protein were isolated to evaluate the efficiency of transfection and determine the appropriate sequence for the next experiments.

### 2.8 Lentiviral Vectors and Cell Transduction

For *SLC7A2* silencing, human *SLC7A2*‐targeting short hairpin RNA (shRNA) was inserted into the hU6‐MCS‐CBh‐gcGFP‐IRES‐puromycin lentiviral vector (GV493, Genechem, China). To induce *SLC7A2* overexpression, complete *SLC7A2* cDNA was inserted into the puromycin lentiviral vector Ubi‐MCS‐3FLAG‐CBh‐gcGFP‐IRES (GV492, Genechem). Precisely, cells (1 × 10^5^ cells/well) were grown in culture plates until they reached a 20% to 30% confluency. A lentivirus was inserted into the cells with a multiplicity of infection (MOI) of 10, via the HiTransG A/P infection‐enhancing solution. Following selection, the cells were maintained in 10% FBS‐enriched DMEM with 2 μg/mL puromycin for 1 week.

### 2.9 Transwell Migration and Invasion Assays

To observe cell migration and invasion, Transwell inserts (8 μm) (Corning, USA) with coated or uncoated Matrigel were used. Approximately 1 × 10^5^ cells were resuspeneded in FBS‐free DMEM (200 μL) and inoculated into the top chamber of the inserts. In the bottom chamber, 600 μL of 15% FBS‐enriched DMEM was added. The cells (non‐migrated) at the top chamber of the inserts were detached via a cotton swab following a 24 h incubation. The migrated cells were placed in 4% PFA for 20 min and stained with 0.1% crystal violet. These migrated cells were quantified by using a microscope.

### 2.10 Evaluation of Micro‐Lymphatic Vessel Density (MLVD)

To evaluate tumor‐related lymphangiogenesis, the MLVD was quantified in tumor sections by detection of the LYVE1‐positive vessels [21]. At low magnification (40×), the most vascularized region within the tumor was detected. These regions were counted (manually) at high magnification (400×). Three random fields were selected to detect a total number of lymphatic vessels, and the resulting count was noted as per unit area. Endothelial staining in non‐endothelial structures and large vessels was not considered in the lymphatic vessel counts.

### 2.11 Flow Cytometry

To examine apoptosis, cells were stained with Annexin V‐FITC/PI Detection Kit (SunGENE Biotech, China). Approximately 1 × 10^5^ cells/well were grown with 90% confluency in six‐well plates. Cells were harvested and processed for Flow cytometric (FCM; BD Biosciences, USA) analysis. For cell cycle analysis, the stably transfected cells were fixed in chilled 70% ethanol at 4°C for 24 h. They were kept with propidium iodide (PI) and RNase A. The results were observed using a flow cytometer.

### 2.12 EdU Proliferation Assay

The EdU detection reagent (RiBoBio, Guangzhou, China) was used to assess cell proliferation. Precisely, cells were cultured with 70 to 80% confluency and kept with 50 μM EdU at 37°C for 2 h. Subsequently, the cells were fixed in 4% PFA solution, stained with Apollo dye for 30 min, and examined using an inverted microscope.

### 2.13 Wound Healing Assay

Approximately 2.5 × 10^5^ cells/well were cultured in a six‐well plate. They were maintained with 60%–70% confluency before being transfected via a liposome transfection kit (NEOFECTTMDNA transfection reagent, China). After 24 h, the exhausted medium was changed and the cells were allowed to grow till 95% confluency. Four scratches were marked in the confluent monolayer with the help of a 200 μL pipette tip. After 24 h culture in FBS‐free DMEM, the cells were observed under an inverted microscope.

### 2.14 In Vivo Model Development

To develop an animal model of HNSCC LM, approximately 5 × 10^6^ cells (OE‐SLC7A2 and Vector groups) were administered into the footpads of nude mice (5–6 weeks old) (Chongqing Tengxin Biotechnology Co, China). Weekly assessments were conducted to monitor the body weights and tumors of the mice. Primary tumors and ipsilateral lymph nodes (LNs) were collected from sacrificed mice after 30 days. For hematoxylin and eosin (H&E) staining, pathological paraffin sections were prepared from LN tissues. The study protocol was approved by the Animal Care and Treatment Committee of the above‐mentioned University.

### 2.15 Immunofluorescence (IF) Assay

Paraffin‐embedded sections of tumor tissue were processed by dewaxing and hydrating, followed by an antigen retrieval step. A 3% BSA solution was dropwise added and blocked for 30 min. The sections were kept in a humidified box with mixed reagents of primary antibodies (Ki‐67, 1:1000, and LYVE‐1, 1:30; GB121141 and GB113499) (Servicebio China) for 24 h at 4°C. The respective secondary antibodies (Goat‐Anti‐Mouse IgG, 1:130 and Goat‐Anti‐Rabbit IgG, 1:400; GB21301 and GB25303, both from Servicebio China) were added on the sections and kept in the dark for 50 min at 25°C. After counterstaining the cell nuclei with DAPI, cells were exposed to autofluorescence quenching agent B solution for 5 min. Anti‐fluorescence quenching mounting agent was used to mount the sections. The images were captured via an upright fluorescence microscope (Nikon Eclipse C1).

### 2.16 Bioinformatics

Differential expression of *SLC7A2* was observed via the Tumor Immune Estimation Resource (http://timer.cistrome.org/). Disease‐free survival (RFS) and *SLC7A2* difference analyses were conducted on HNSCC in the TCGA database accessible via the public website (http://gepia2.cancer‐pku.cn). The STRING database (https://cn.string‐db.org/) was used to construct protein–protein interaction (PPI) networks for genes associated with *SLC7A2*. The functions related to differential genes and the pathways involved were annotated based on Gene Ontology (http://pgrc.ipk‐gatersleben.de/misa/) and KEGG databases (http://emboss.sourceforge.net/). The Cancer Genome Atlas (TCGA) provided the HNSCC high‐throughput sequencing datasets (https://portal.gdc.cancer.gov/).

### 2.17 Statistical Analysis

The findings of the study were statistically examined via the SPSS 22 (IBM Corp, USA) and GraphPad Prism, 8.0 (USA). All values are expressed as mean ± standard deviation (SD). The Pearson *χ* ^2^ test was used to determine the correlation between *SLC7A2* expression and clinicopathological characteristics of HNSCC patients, whereas Kaplan–Meier survival curves were used to assess the association between *SLC7A2* expression and five‐year all‐cause mortality in patients with HNSCC. Student's *t*‐tests were used to compare differences between two groups, whereas one‐way ANOVAs followed by Tukey's post hoc tests were used to compare multiple groups. Each experiment was independently repeated at least three times and *p*‐value < 0.05 was considered significant.

## 3 Results

### 3.1 Decreased *SLC7A2* Expression Is Correlated With Poor Prognosis and LNM in HNSCC

**FIGURE 1:**
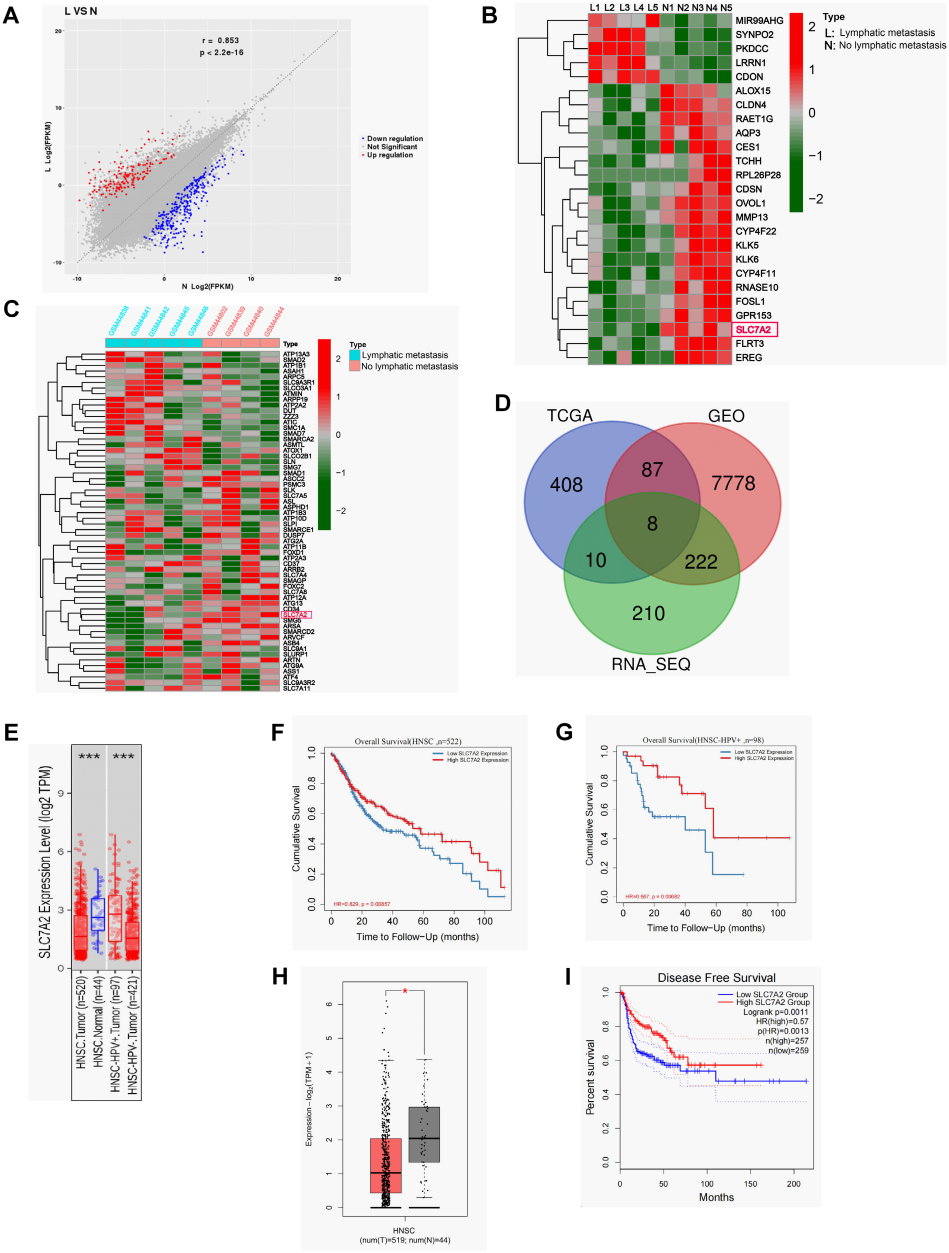
Whole‐transcriptome sequencing of HNSCC tissue and bioinformatics analysis of public databases. (A) Differential genes use scatter plots to display results. The FC threshold for FC was set to |log2FC| > 2, *p* < 0.05. (B) *SLC7A2* has significant differences among differential genes. L: Tissues of HNSCC with LNM; N: Tissues of HNSCC without LNM. (C) Gene expression microarray data were downloaded from the GEO database and *SLC7A2* was displayed in a heat map. The columns represent sample numbers and the rows represent genes. (D) An integrated analysis of these three lists of DEGs revealed *SLC7A2* co‐DEGs. (E) Expression levels of *SLC7A2* in HPV‐positive and HPV‐negative HNSCC. (F) Examination of *SLC7A2* survival in HNSCC. (G) Examination of *SLC7A2* survival in HPV‐positive HNSCC. (H) Transcription of *SLC7A2* in 519 cases of HNSCC and 44 normal tissues. (I) Analysis of *SLC7A2* disease‐free survival in HNSCC. *p ≤ 0.05, **p ≤ 0.01, ***p ≤ 0.001.

The surgically resected primary tumor tissues of 10 HNSCC patients (five with LNM and five without) were subjected to whole‐transcriptome sequencing (relevant data are in the attachment). Using a log2 FC threshold of 2 (|log2 (FC)| > 2, *p*‐value < 0.05), 530 differentially expressed genes (DEGs) were identified, with 206 upregulated and 324 downregulated genes (Figure 1A). Notably, SLC7A2 is one of the genes with obvious differential expression (Figure 1B). Microarray data from five HNSCC cases with LN metastasis and four cases without LN metastasis were retrieved from the GEO database to screen for differentially expressed genes (*p* < 0.05) (Figure 1C). Additionally, the HNSCC transcripts from the TCGA database were downloaded for analysis. The comparison of differentially expressed genes between groups with and without LNM across three datasets revealed SLC7A2 as a co‐expressed gene with a consistent expression pattern (Figure 1D). Moreover, an extensive pan‐cancer analysis was conducted using the TIMER database to assess *SLC7A2* levels across 33 prominent human cancers. The expression level of SLC7A2 is statistically higher in normal tissues (*n* = 44) compared with HNSCC tissues (*n* = 520), and the level of *SLC7A2* was upregulated in HPV‐positive HNSCC (*n* = 97) than that in HPV‐negative HNSCC (*n* = 421) (Figure 1E). Further, the high levels of *SLC7A2* were correlated with prolonged survival in HNSCC patients (*p* ≤ 0.05) (Figure 1F). This survival difference was even more prominent in HPV‐positive patients (*p* ≤ 0.006) (Figure 1G). Additionally, based on TCGA data analysis, the level of *SLC7A2* in HNSCC was substantially decreased in contrast to normal tissues, and low *SLC7A2* levels were related to poor DFS (Figure 1H,I). Collectively, these outcomes suggested that *SLC7A2* is differentially expressed in HNSCC with and without LNM, and HNSCC patients with low *SLC7A2* expression have reduced survival, suggesting that *SLC7A2* could be a possible prognostic factor.

### 3.2 *SLC7A2* Expression Was Down‐Regulated in HNSCC Tissue Samples, Which May Be a Risk Factor for the LNM of HNSCC

**FIGURE 2:**
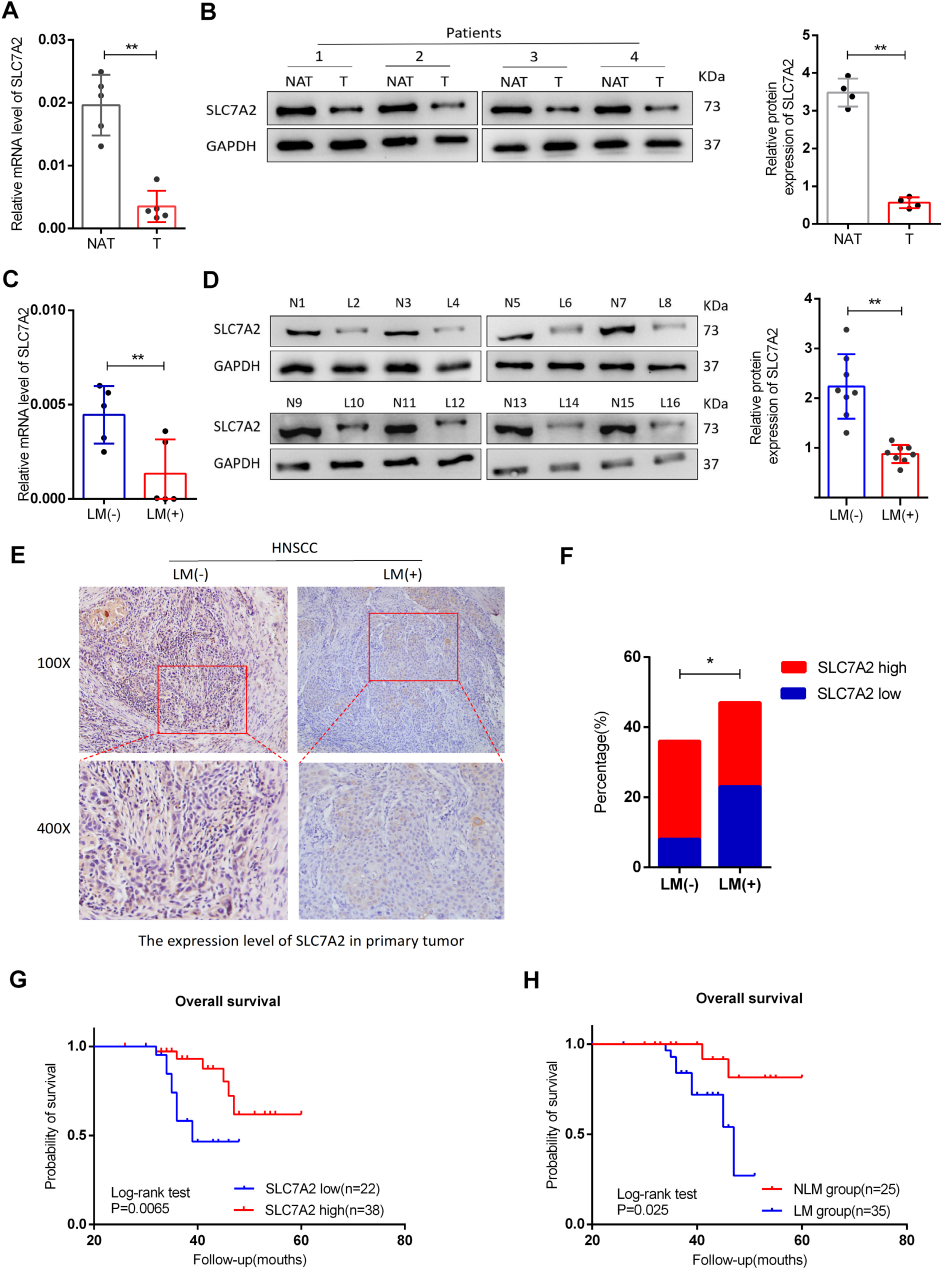
(A) *SLC7A2* mRNA levels in adjacent normal mucosal tissue and HNSCC tissue. (B) Expression of *SLC7A2* protein in adjacent normal mucosal tissue and HNSCC (NAT; adjacent normal mucosal tissue, and T depicts HNSCC tissue). (C) Expression of *SLC7A2* mRNA in HNSCC tissue without LNM and HNSCC tissue with LNM. LM (−) depicts without LNM, and LM (+) depicts with LNM. (D) SLC7A2 protein levels in HNSCC tissue without LNM and HNSCC tissue with LNM. (E) Expression of *SLC7A2* in 79 cases of HNSCC tissues, magnification divided into 100 and 400×. (F) Low and high expression ratios of *SLC7A2* in 79 cases of HNSCC. (G) Comparisons of overall survival between low *SLC7A2* expression group and high *SLC7A2* expression group. (H) Comparisons of overall survival between NLM group and LM group.

For further detection, we quantitatively determined the mRNA concentrations of *SLC7A2* in healthy mucosa (*n* = 5) and HNSCC tissues (*n* = 5), and protein levels of *SLC7A2* in healthy mucosa (*n* = 4) and HNSCC tissues (*n* = 4). The results showed that *SLC7A2* mRNA and protein levels were reduced in HNSCC compared with the normal mucosa group (Figure 2A,B). In addition to analyzing 10 cases of HNSCC sample tissues (five cases of LNM and five cases without LNM) using qRT‐PCR, we also conducted Western blot detection and analysis on 16 cases of HNSCC sample tissues (eight cases of LNM and eight cases without LNM). Notably, the *SLC7A2* mRNA and protein levels were found to be significantly lower in the HNSCC group with LNM compared to the group without LNM (Figure 2C,D). Tissues obtained from 79 HNSCC patients were stained via IHC. The findings demonstrated that the patients with LNM had a higher incidence of weak staining than those without LNM (23/43 vs. 8/36; *p* < 0.01) (Figure 2E,F; Table 1). The *SLC7A2* expression is significantly correlated with LNM and clinical stage in HNSCC patients (Table 2). Further, the outcomes of the univariate and multivariate analysis also demonstrated that the level of *SLC7A2* was a substantial indicator of HNSCC (Table 3). Finally, survival analysis showed that elevated *SLC7A2* levels corresponded to improved overall survival of HNSCC patients (Figure 2G), and patients with LNM had shorter survival times (Figure 2H). Overall, these results suggest that *SLC7A2* expression level may be directly related to clinical outcomes and lymphatic metastasis.

**TABLE 1:** The immunohistochemical expression of SLC7A2 in clinical specimens of patients.

Tissue	Expression of SLC7A2		Number of patients	*p* *
	Negative	Positive		
Primary tumor in patients without lymphatic metastasis	8	28	36	0.006
Primary tumor in patients with lymphatic metastasis	23	20	43	
Metastatic lymph node in patients	31	48	79	

* *p*‐values are from *χ* ^2^ test or Fisher's exact test and were statistically significant when < 0.05.

**TABLE 2:** Correlation between SLC7A2 expression and clinicopathological characteristics of HNSCC patients.

Characteristics	No. of case (%)	SLC7A2 expression		*χ* ^2^	*p*
		Low (*N* = 31)	High (*N* = 48)		
Gender					
Male	76 (96.2)	30	46	0.046	0.66
Female	3 (3.8%)	1	2		
Age (y)					
< 60	30 (38)	8	22	3.207	0.059
≥ 60	49 (62)	23	26		
BMI					
< 18	11 (13.9)	3	8	0.768	0.299
≥ 18	68 (86.1)	28	40		
Cigarette					
Yes	72 (91.1)	29	43	0.367	0.431
No	7 (8.9)	2	5		
Alcohol					
Yes	52 (65.8)	20	32	0.039	0.516
No	27 (34.2)	11	16		
LN metastasis					
Yes	43 (54.4)	23	20	8.034	0.006**
No	36 (45.6)	8	28		
Clinical stage					
Early	22 (27.8)	3	19	8.384	0.004**
Middle and late stage	57 (72.2)	28	29		
Pathologic differentiation					
Well‐moderate	73 (92.4)	29	44	0.095	0.561
Poor	6 (7.6)	2	4		

** *p* < 0.01.

**TABLE 3:** Univariate and multivariate Cox regression analyses of overall survival in HNSCC patients.

Variables	Univariate analysis			Multivariate analysis		
	*p*	Hazard ratio	95% confidence interval	*p*	Hazard ratio	95% confidence interval
SLC7A2	0.01*	3.74	1.31–10.64	0.03*	3.45	1.12–10.60
Gender	0.82	0.05	0–387			
Age (y)	0.38	1.59	0.56–4.45			
BMI	0.58	0.66	0.15–2.95			
Cigarette	0.56	1.83	0.23–14.12			
Alcohol	0.86	0.92	0.33–2.53			
LN metastasis	0.05*	0.27	0.07–0.99	0.83	1.20	0.22–6.39
Clinical stage	0.02*	0.16	0.03–0.72	0.05*	0.16	0.03–1.01
Pathologic differentiation	0.94	1.06	0.24–4.67			

* *p* < 0.05.

### 3.3 *SLC7A2* Upregulation May Inhibit HNSCC Cell Propagation, Migration, and Invasion In Vitro

**FIGURE 3:**
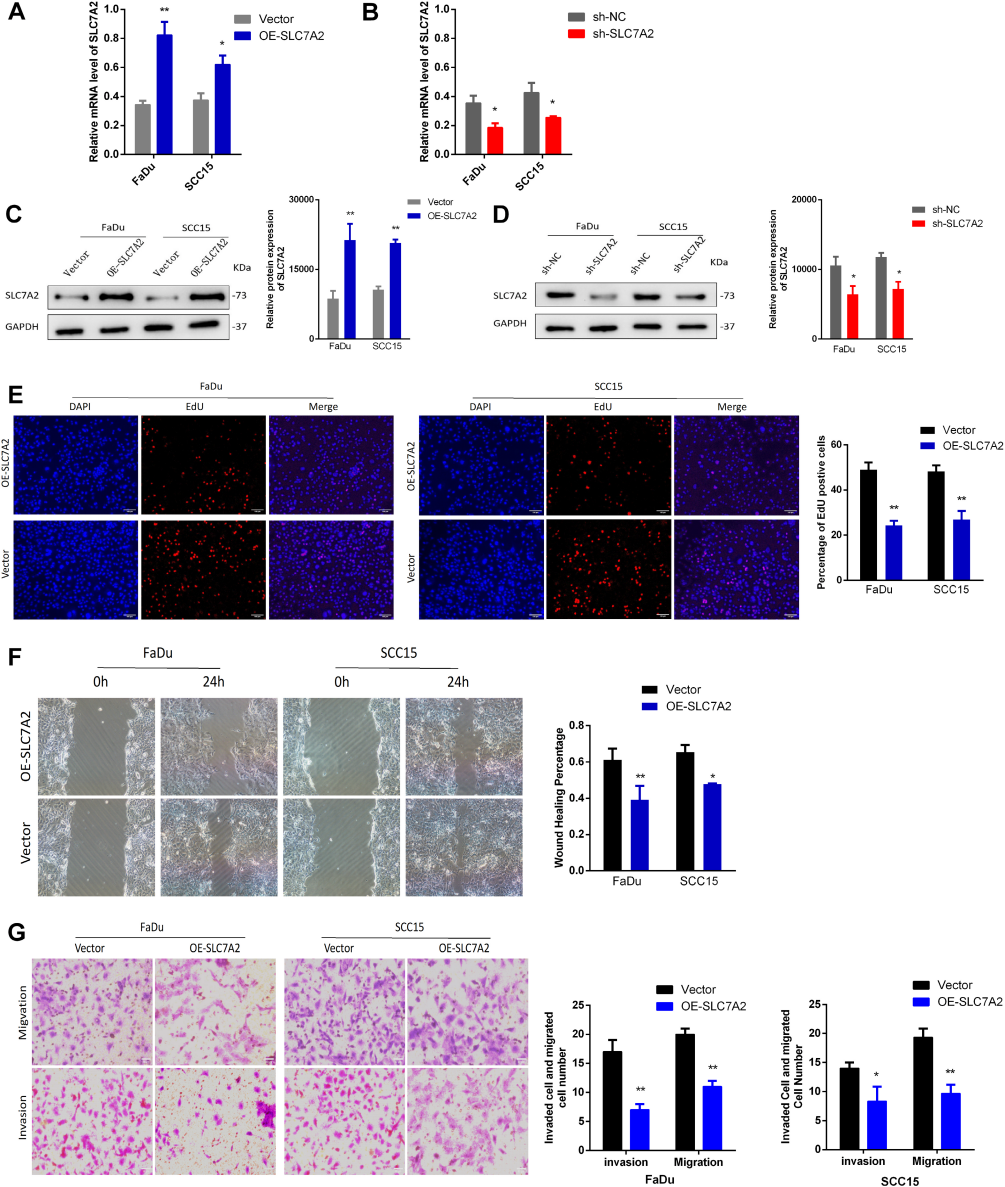
*SLC7A2* affects HNSCC cell propagation in vitro. (A) The mRNA level of *SLC7A2* in *SLC7A2* overexpression HNSCC cells. (B) The mRNA level of *SLC7A2* in *SLC7A2* knockdown HNSCC cells. (C) The protein level of *SLC7A2* in *SLC7A2* overexpression HNSCC cells. (D) The protein level of *SLC7A2* in *SLC7A2* knockdown HNSCC cells. (E) EdU assay to evaluate the cell proliferation of HNSCC cell lines after overexpression of *SLC7A2*. (F) Wound healing analysis of OE‐*SLC7A2* HNSCC cell. (G) Transwell test and the analysis results of OE‐*SLC7A2* HNSCC cell.

To assess the mode of action of *SLC7A2* in HNSCC, cell experiments were performed. Initially, *SLC7A2* was inhibited and overexpressed using lentiviral shRNA and an overexpression vector in FaDu and SCC15 cells. Subsequently, qRT‐PCR and WB were performed to quantify *SLC7A2* mRNA and protein levels, respectively. The results indicated a substantial upregulation in the overexpression group (OE‐*SLC7A2*) and a reduction in the knockdown group (sh‐*SLC7A2*) relative to the negative control group (sh‐NC and vector) (Figure 3A–D). Further, an EdU cell proliferation assay was performed on FaDu and SCC15 cells stably overexpressing *SLC7A2*, showing reduced proliferative ability in the *SLC7A2* overexpressing cells in contrast to the control group (Figure 3E). It was further confirmed by scratch assay and Transwell experiments that *SLC7A2* overexpression inhibited the cell migration and invasion (Figure 3F,G).

**FIGURE 4:**
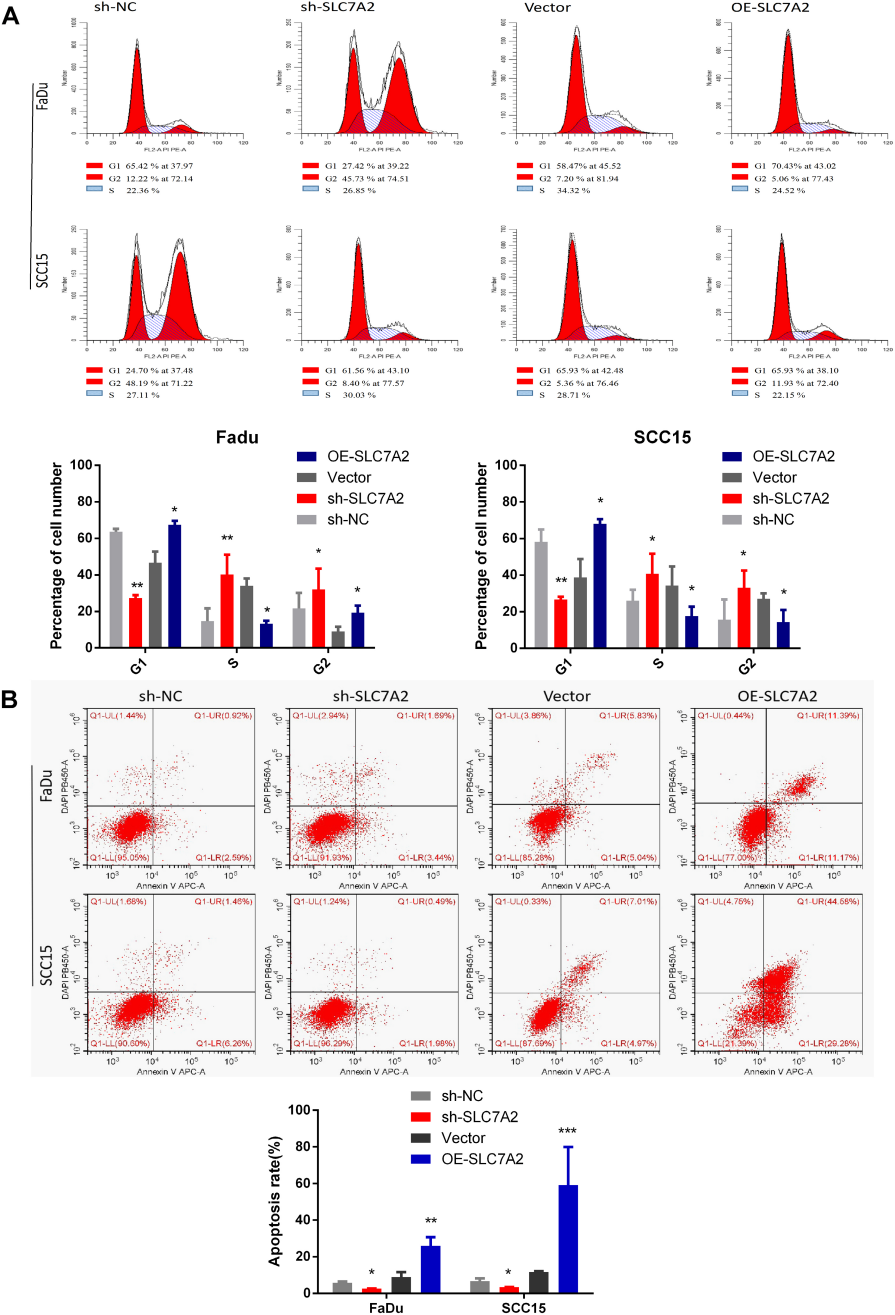
*SLC7A2* affects the HNSCC cell cycle and apoptosis in vitro. (A) Cell cycle detection results and statistical analysis in knockdown and overexpression of *SLC7A2* HNSCC cell lines. (B) Results and statistical analysis of cell apoptosis in knockdown and overexpression of *SLC7A2* HNSCC cell lines.

The cell cycle and apoptosis of HNSCC cells exhibiting overexpressed or knocked down *SLC7A2* were evaluated by flow cytometry. The findings indicated that the quantity of S phase cells in the OE‐*SLC7A2* cell line was diminished in contrast to the control group, while the number of S phase cells in the sh‐*SLC7A2* cell line was found to be elevated than that of the NC group. Besides, cell apoptosis was observed to be lower in the sh‐*SLC7A2* cell line in contrast to the NC group, whereas it was upregulated in the OE‐*SLC7A2* cell line in contrast to the control group (Figure 4A,B). Overall, it was determined that *SLC7A2* promoted apoptosis while suppressing the HNSCC cell propagation, migration, and invasion in vitro.

### 3.4 *SLC7A2* Upregulation Inhibits Lymphangiogenesis in HNSCC Cells

**FIGURE 5:**
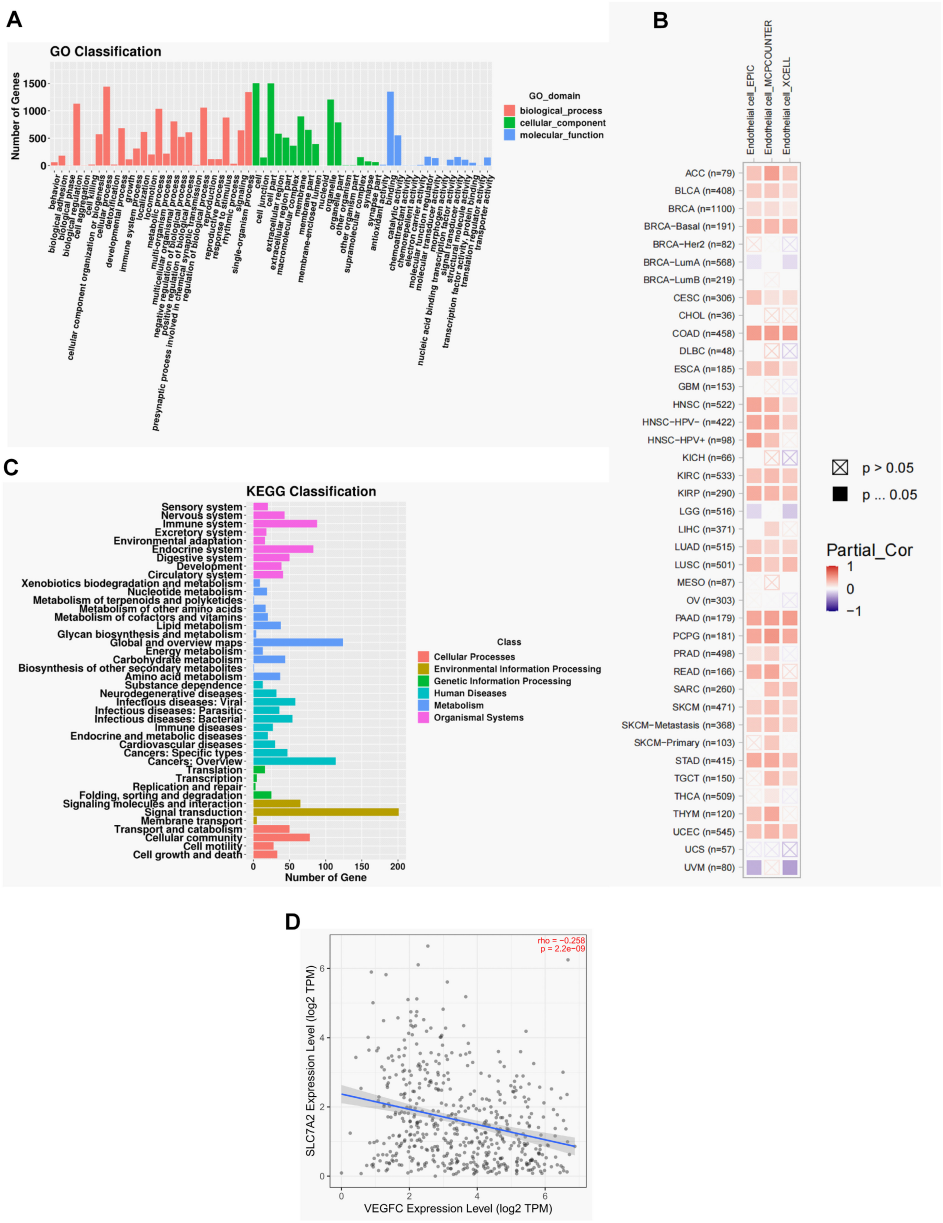
Bioinformatics analysis of *SLC7A2*. (A) GO enrichment analysis. (B) *SLC7A2* and endothelial cell infiltration analysis. (C) KEGG enrichment analysis. (D) Correlation analysis between *SLC7A2* and *VEGFC*.

**FIGURE 6:**
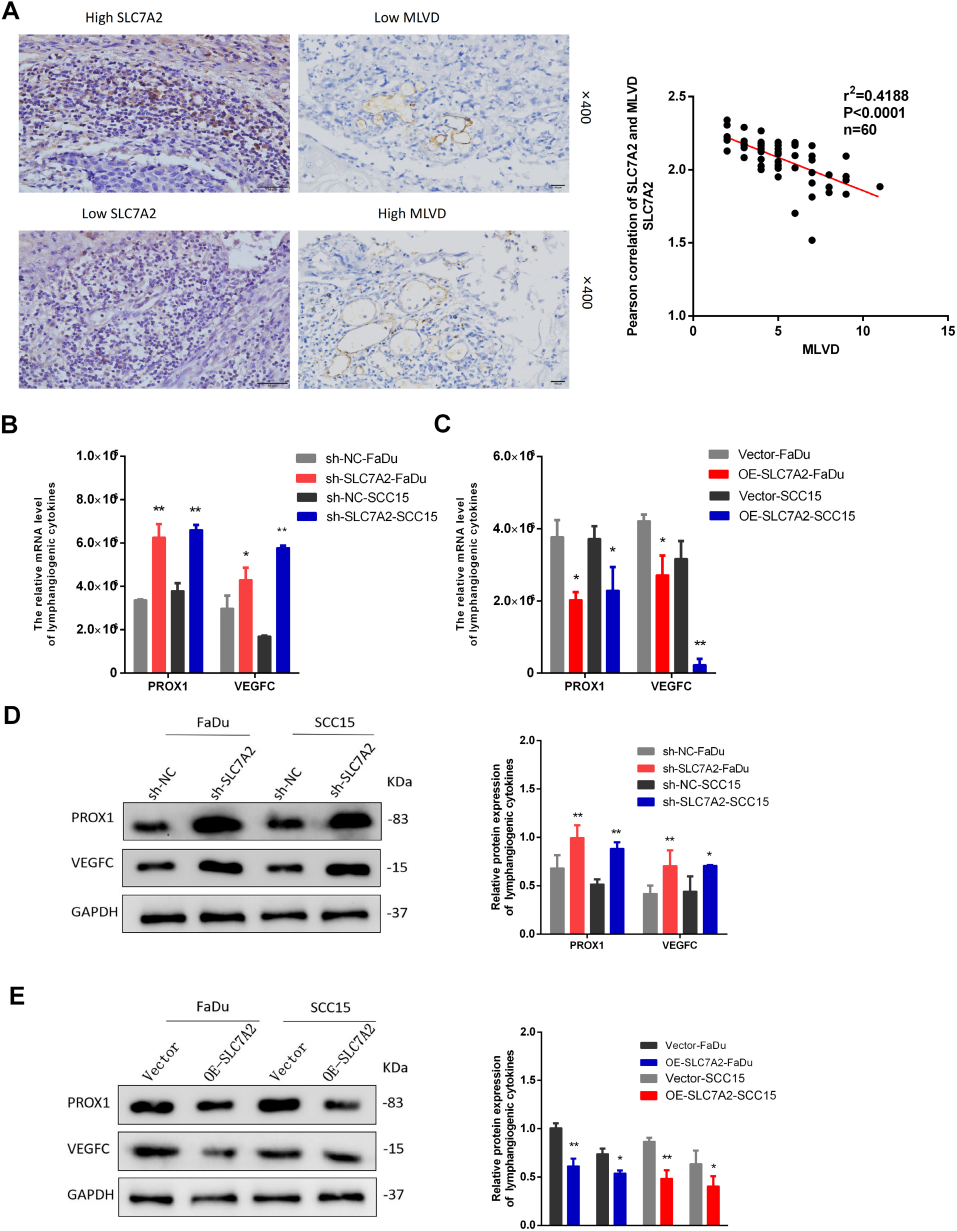
The relationship between *SLC7A2* and lymphangiogenesis in HNSCC tissues and its effect on lymphangiogenesis in vitro. (A) Immunohistochemical staining detects a negative correlation between *SLC7A2* and MLVD in HNSCC tissues. (B) The mRNA level of lymphangiogenic factor (PROX1, VEGFC) after knockdown *SLC7A2* in HNSCC cells. (C) The mRNA level of lymphangiogenic factor (PROX1, VEGFC) after overexpression *SLC7A2* in HNSCC cells. (D) The protein expression levels of lymphangiogenic factor (PROX1, VEGFC) after knockdown *SLC7A2* in HNSCC cells. (E) The protein expression levels of lymphangiogenic factor (PROX1, VEGFC) after overexpression *SLC7A2* in HNSCC cells.

Tumor lymphangiogenesis is a complex interaction among endothelial cells, tumor cells, and the tumor microenvironment [22, 23]. Our study found that *SLC7A2* inhibits HNSCC propagation, migration, and invasion. In order to identify functional enrichment of differential genes obtained through sequencing, differential gene enrichment analysis using GO and KEGG databases was conducted to determine the primary biological functions and pathways associated with the differential genes (Figure 5A,C). Additionally, TIMER2 database analysis indicated a positive link between *SLC7A2* and endothelial cell infiltration (Figure 5B), as well as a significant negative correlation with the lymphangiogenic factor VEGFC (Figure 5D). The results exhibited that *SLC7A2* might be contributed to the regulation of immune cell infiltration and lymphangiogenesis. Tissue samples from 60 HNSCC patients were analyzed to further evaluate the effect of *SLC7A2* on lymphangiogenesis. Serial sectioning and immunohistochemical staining for LYVE1 were performed to assess tumor‐associated lymphangiogenesis by measuring MLVD, and its relationship with *SLC7A2* expression was examined. The findings suggested that *SLC7A2* expression was inversely related to the MLVD (Figure 6A). The expression of lymphangiogenic cytokines, including VEGFC and PROX1, in HNSCC cell lines with and without *SLC7A2* overexpression and knockdown was evaluated via qRT‐PCR and WB analyses. The findings revealed that the mRNA and protein levels of VEGFC and PROX1 were upregulated in HNSCC after *SLC7A2* knockdown relative to the control group (Figure 6B,D). Conversely, the expression of VEGFC and PROX1 mRNA and protein levels decreased following the overexpression of *SLC7A2* (Figure 6C,E). In vitro analysis indicated that *SLC7A2* inhibits lymphangiogenesis in HNSCC.

### 3.5 *SLC7A2* May Inhibit Lymphangiogenesis in HNSCC Cells by Regulating CPB2 Expression

**FIGURE 7:**
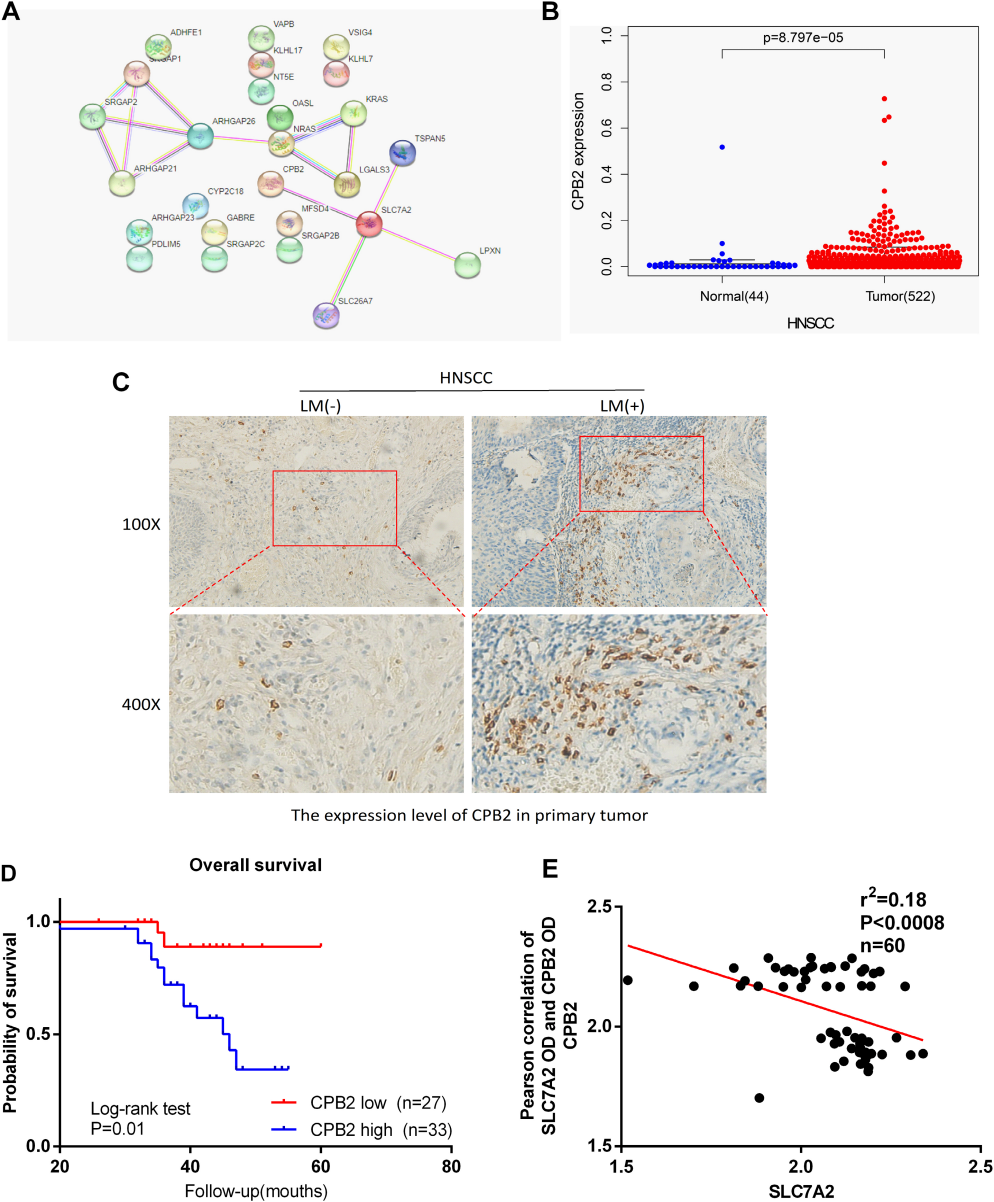
(A) SLC7A2 protein‐related function network, related to CPB2; (B) Transcription of CPB2 in 522 cases of HNSCC and 44 normal tissues. (C) Expression of CPB2 in 60 cases of HNSCC tissues, magnification divided into 100 and 400×. (D) Comparisons of overall survival between low CPB2 expression group and high CPB2 expression group. (E) Immunohistochemical staining detects a negative correlation between *SLC7A2* and CPB2 in HNSCC tissues.

**FIGURE 8:**
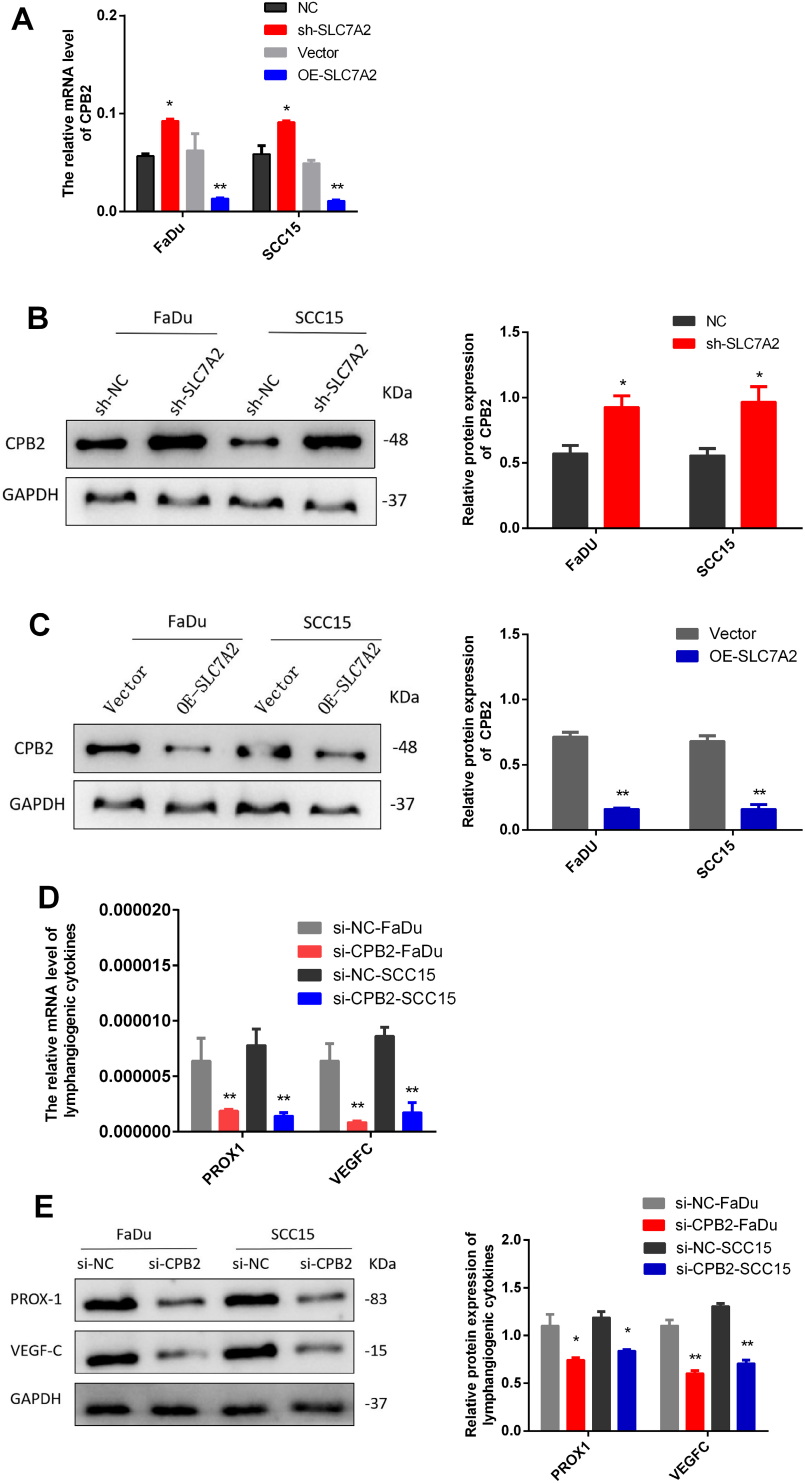
(A) The mRNA level of CPB2 after knockdown and overexpression of *SLC7A2* in HNSCC cells. (B) The protein expression levels of CPB2 after knockdown of *SLC7A2* in HNSCC cells. (C) The protein expression levels of CPB2 after overexpression of *SLC7A2* in HNSCC cells. (D) The mRNA level of lymphangiogenic factor (PROX1, VEGFC) after small interference of CPB2 in HNSCC cells. (E) The protein expression levels of lymphangiogenic factor (PROX1, VEGFC) after small interference of CPB2 in HNSCC cells.

Based on TCGA data, a PPI network was generated to examine *SLC7A2*'s downstream mechanism. The analysis revealed a relationship between *SLC7A2* and CPB2 level (Figure 7A). Transcriptome data from the TCGA database was used to analyze the expression of CPB2 in HNSCC. The study revealed higher CPB2 expression in tumor tissue compared to normal mucosal tissue (Figure 7B). Subsequent immunohistochemical analysis of 60 HNSCC tissue samples, based on clinical characteristics, showed higher CPB2 expression in tissues with LNM than those without (Figure 7C). Patients with high CPB2 expression had lower survival times (Figure 7D). Furthermore, a negative correlation between *SLC7A2* and CPB2 expression was observed in the same sample tissue (Figure 7E). The expression of CPB2 was quantified in *SLC7A2* knockdown and overexpressing HNSCC cell lines to find the regulatory effect of *SLC7A2* on CPB2. The outcomes exhibited the upregulation of CPB2, mRNA, and protein levels in *SLC7A2* knockdown cells, while downregulation was observed in *SLC7A2* overexpressed cells (Figure 8A–C). Additionally, the involvement of CPB2 in the lymphangiogenesis of HNSCC was also validated through the development of si‐CPB2‐NC and si‐CPB2 HNSCC cell lines, followed by an evaluation of the mRNA and protein levels of VEGFC and PROX1 in HNSCC. In contrast to the si‐CPB2‐NC group, the si‐CPB2 group demonstrated decreased levels of PROX1 and VEGFC factors at gene and protein levels. This finding suggests that inhibiting CPB2 can impede lymphangiogenic signals (Figure 8D,E). Collectively, this study provides evidence that *SLC7A2* may inhibit lymphangiogenesis in HNSCC via the regulation of CPB2 functions.

### 3.6 In Vivo, *SLC7A2* Upregulation Suppresses Tumor Cell Growth and Lymphatic Metastasis

**FIGURE 9:**
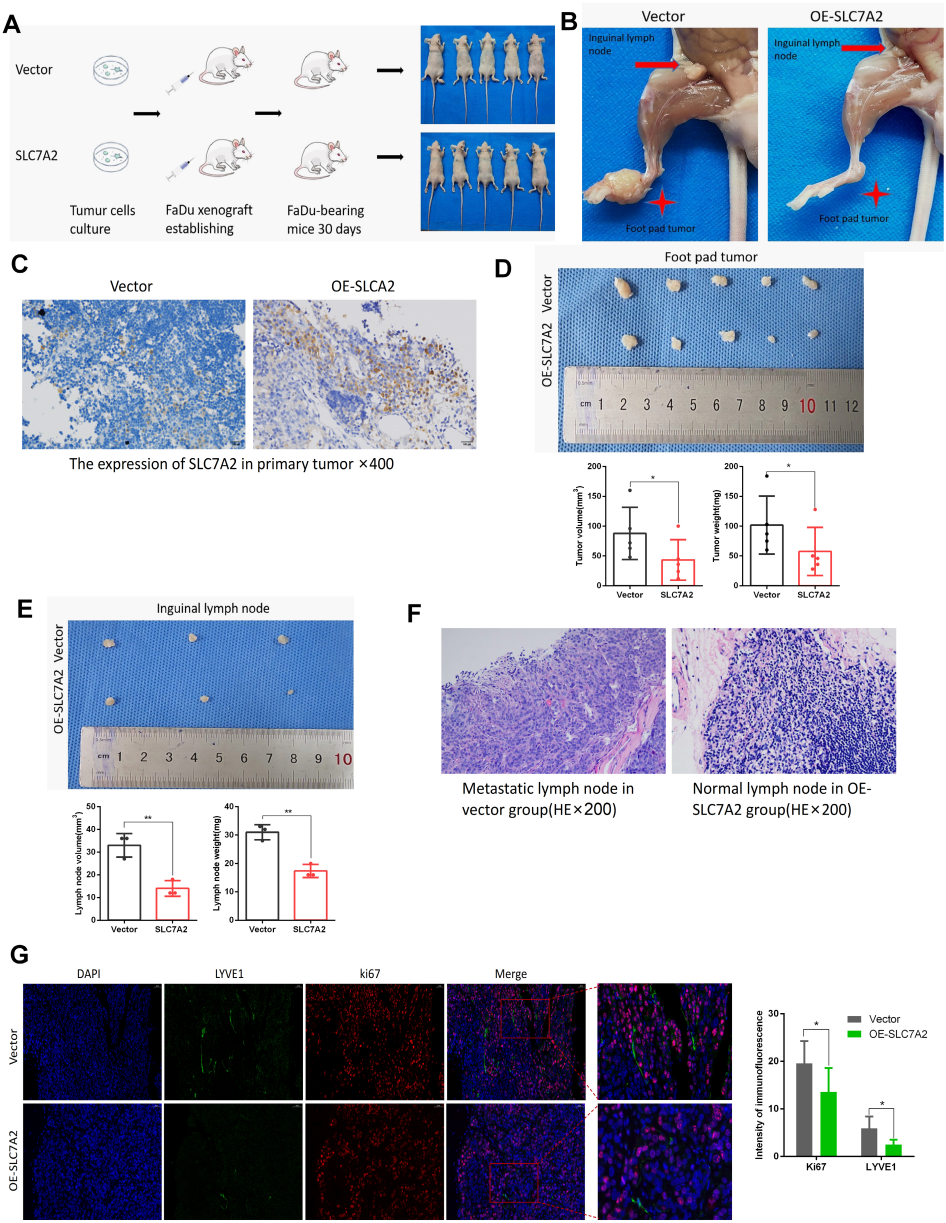
*SLC7A2* overexpression suppresses the tumor cell growth and lymphatic metastasis in vivo. (A) FaDu cells overexpressing *SLC7A2* were administered into the footpads of nude mice and observed for 30 days before being sacrificed. (B) Images of primary tumors and inguinal lymph node of nude mice. (C) The expression of *SLC7A2* in the primary tumors. (D) Images of primary tumors in nude mice and analysis of results. (E) Images of inguinal lymph node in nude mice and analysis of results. (F) HE staining pictures of inguinal lymph nodes in nude mice. (G) Immunofluorescence double staining to detect the Ki67 (red) and LYVE1 (green) expressions in primary tumors of nude mice and statistical pictures.

In vivo, findings revealed the effects of *SLC7A2* in the regulation of LNM. To develop an LNM model of HNSCC cells, FaDu cells transfected with an empty vector or a *SLC7A2* overexpressing lentivirus were xenografted into the footpads of nude mice. All mice were euthanized after 30 days, and their footpads tumor and inguinal lymph node were harvested (Figure 9A,B). Meanwhile, we measured and analyzed the number, volume, and weight of footpad tumors and inguinal metastatic lymph nodes, immunohistochemical staining to detect the expression of *SLC7A2* in tumors, and HE staining to determine whether inguinal lymph nodes had metastasized. The results showed that the footpad tumor tissues of five pairs of mice were harvested in this experiment, and IHC confirmed that the expression of *SLC7A2* in the OE‐*SLC7A2* group was higher than that in the vector group (Figure 9C). The growth rate of primary tumors in the OE‐*SLC7A2* group was slower than that in the Vector group (Figure 9D). At the same time, the inguinal lymph nodes of three pairs of mice were harvested. The volume of the lymph nodes of the three mice in the OE‐*SLC7A2* group was smaller than that of the Vector group (Figure 9E), and no LNM occurred, while three mice in the Vector group developed LNM (Figure 9F). Furthermore, immunofluorescence double staining was conducted on footpad tumor tissues to identify Ki67 and LYVE1. The results indicated that the overexpression group possessed footpad tumors with reduced expression of Ki67 and LYVE1 in comparison with the empty group (Figure 9G). These results indicated that upregulation of *SLC7A2* expression inhibits tumor growth and LNM in mice, and may inhibit tumor cell proliferation and lymphangiogenesis in vivo.

## 4 Discussion

HNSCC is the most common cancer of the head and neck region. Despite therapeutic advancements, the rates of mortality and recurrence continue to be elevated, mainly during the later stages. Its survival rate has not exhibited substantial improvements [24, 25]. The head and neck region has unique anatomical features, with the presence of a large number of LNs. This region contains around 150 to 300 LNs, which accounts for nearly half of the total LNs in the human body (i.e., 500 to 600 LNs) [26]. They are crucial in the progression of metastatic tumors, serving as hubs for signaling pathways such as cell death, propagation, immune regulation, and secondary metastasis to distant areas via various mechanisms [27]. LNM is a significant independent prognostic factor and a clinically prominent characteristic of advanced HNSCC. Currently, the study of molecular pathways is an essential initial step due to the absence of efficacious LNM‐targeted therapeutic approaches. Thus, this study presented new insights into targeted therapy for tumors by examining the key variables and mechanisms related to HNSCC lymphatic metastasis.

Whole‐transcriptome sequencing is a highly accurate and efficient technology used for detecting differential genes and disease‐related biomarkers [28]. In this study, we initially employed whole‐genome transcriptome sequencing to identify differentially expressed genes in HNSCC tissues, distinguishing between those with and without LNM. Notably, the *SLC7A2* gene emerged as one of the most significantly differentially expressed. By comparing data from the Gene Expression Omnibus (GEO) and The Cancer Genome Atlas (TCGA), we further validated the underexpression of S*LC7A2* in HNSCC through bioinformatics analysis. Specifically, in HPV‐negative HNSCC samples, the expression level of *SLC7A2* was found to be even lower. These findings suggest that the reduced expression of *SLC7A2* is closely associated with the poor prognosis of HNSCC patients, indicating that it may be a key gene influencing LNM in this context.

The transportation of cationic amino acids, including lysine and L‐arginine, into cells is facilitated by the membrane protein *SLC7A2* [29]. It potentially exerts regulatory effects on metastatic tumor development by activating multiple signaling cascades [30]. It may reduce the levels of CXCL1, resulting in the suppression of myeloid‐derived suppressor cell penetration and immune evasion in HCC [16]. Ovarian cancer cells exhibit enhanced migration and invasion capabilities when *SLC7A2* expression is downregulated due to genetic variation [17]. In non‐small cell lung cancer, *SLC7A2* also functions as a tumor suppressor by regulating drug sensitivity, immune infiltration, and cell survival [31]. The results of this study indicate that the overexpression of *SLC7A2* significantly promotes apoptosis in HNSCC cells while effectively inhibiting their growth, proliferation, and metastatic capabilities. These findings align with the established role of *SLC7A2* in various cancer types, suggesting that it may function as a tumor suppressor gene across multiple tumors. *SLC7A2* likely exerts its anti‐tumor effects by regulating cell cycle‐related proteins or influencing specific signaling pathways. Additionally, the overexpression of *SLC7A2* may modify certain factors within the tumor microenvironment, thereby inhibiting tumor invasion and metastasis.

This study used the STRING protein interaction network tool to find possible targets of *SLC7A2*. The top five interacting proteins were identified, among them, CPB2 was the main interactive protein. It is also referred to as a human TAFI. It has been identified as an abnormally expressed protein in various malignancies. Notably, in breast cancer, CPB2 expression is positively correlated with lymphovascular invasion. It is also regarded as a prognostic indicator for human cancer [19, 20]. Recent findings have demonstrated that the plasminogen activation system is a critical regulator in cancer cells’ invasion and metastasis, which are dependent on extracellular matrix degradation during local angiogenesis [32, 33]. In this study, we investigated the molecular mechanisms underlying LNM in head and HNSCC and proposed a hypothesis that *SLC7A2* may influence lymphangiogenesis by regulating the expression levels of CPB2. To test this hypothesis, we first analyzed the expression of CPB2 in HNSCC tissues, noting a significant increase in cases with LNM. Additionally, we observed a negative correlation between the expression of CPB2 and the levels of *SLC7A2*, which preliminarily supports our hypothesis. Furthermore, we conducted overexpression and knockdown experiments of *SLC7A2* in HNSCC cell lines and monitored the effects of these treatments on CPB2 expression. The results indicated that overexpression of *SLC7A2* significantly reduced CPB2 expression, thereby inhibiting its function, while the knockdown of *SLC7A2* led to an upregulation of CPB2 levels. These findings further confirm that *SLC7A2* exerts a direct regulatory effect on CPB2 expression. These discoveries not only offer a new perspective for understanding the molecular mechanisms of LNM in HNSCC but also establish a theoretical foundation for the development of potential therapeutic strategies targeting *SLC7A2* and CPB2.

We focused on the role of lymphatics in tumor metastasis and their regulatory mechanisms. Evidence indicates that lymphatics are not only involved in the metastatic process of tumors [34], but also that tumor‐induced lymphangiogenesis exhibits characteristics that suppress the immune response, thereby creating favorable conditions for the colonization and metastasis of tumor cells [35]. Lymphangiogenesis is a complex regulatory process driven by molecules such as VEGFC and D‐VEGFR3 [36], with PROX1 serving as a core regulatory factor that promotes the formation of lymphatics by activating the expression of VEGFR3, thereby establishing a positive feedback loop that sustains lymphangiogenesis [37]. Our study found that in HNSCC tissue samples, the expression of *SLC7A2* is negatively correlated with lymphatic density, and the overexpression of *SLC7A2* can reduce the expression levels of lymphangiogenesis‐related factors. In vivo experiments further confirmed that the upregulation of *SLC7A2* can inhibit LNM, tumor growth, and the formation of tumor‐related lymphatics. Additionally, by constructing a Si‐CPB2 HNSCC cell model, we observed that the interference of CPB2 diminished the lymphangiogenesis signal, indicating that *SLC7A2* may influence lymphangiogenesis by regulating CPB2. Although this study may be constrained by its sample size and individual differences, future research will investigate the mechanisms of action of *SLC7A2* and its interactions with other genes. This work aims to assess *SLC7A2*'s role in LNM in HNSCC and to provide new perspectives for diagnosis and monitoring. Furthermore, the research will focus on developing small molecule modulators that target *SLC7A2* to enhance its capacity to inhibit tumor metastasis. Concurrently, combination treatment strategies, including immunotherapy and radiotherapy, will be explored to improve therapeutic outcomes for HNSCC patients and to introduce new possibilities for treatment.

## 5 Conclusion

This study provides innovative insights into the inhibitory role of *SLC7A2* in LNM of HNSCC and suggests a mechanism by which it may impede lymphangiogenesis through the modulation of CPB2 activity. Moreover, the expression levels of *SLC7A2* exhibit potential as a key prognostic biomarker for HNSCC, offering a new genetic target for the treatment of LNM in this cancer type. These findings have significant implications for the advancement of personalized cancer therapy and lay a solid foundation for the development of future clinical treatment strategies.

## Author Contributions

**Kai Song:** data curation (equal), software (equal), validation (equal), writing – original draft (equal). **Yanshi Li:** resources (equal), software (equal), validation (equal). **Kai Yang:** data curation (supporting). **Tao Lu:** methodology (supporting), software (supporting). **Min Wang:** methodology (supporting), software (supporting). **Zhihai Wang:** methodology (supporting), resources (supporting). **Chuan Liu:** methodology (supporting), resources (supporting). **Ming Yu:** formal analysis (supporting). **Mengna Wang:** methodology (supporting). **Zhaobo Cheng:** methodology (supporting). **Min Pan:** resources (supporting), supervision (supporting). **Guohua Hu:** conceptualization (equal), funding acquisition (equal), resources (equal), supervision (equal).

## Ethics Statement

The study was supported by the ethics committee of the first affiliated hospital of Chongqing Medical University and the Animal Care and Treatment Committee of Chongqing Medical University.

## Consent

All the authors agreed to publish the study in the journal.

## Conflicts of Interest

The authors declare no conflicts of interest.

## Supporting information


Data S1.

## Data Availability

All data from this study were used for the publication of this article and are guaranteed for availability.
