# Supplementary material for: Regulatory Effects of SLC7A2‐CPB2 on Lymphangiogenesis: A New Approach to Suppress Lymphatic Metastasis in HNSCC
Source: Cancer Med. 2024 Oct 9;13(19):e70273. doi: 10.1002/cam4.70273 (PMC11468304; doi:10.1002/cam4.70273)
Supplement: Supplementary file 1 — Data S1. [file CAM4-13-e70273-s001.docx]

1.About Reading: There are 10 samples sequenced, and the sequencing results and mapped genes are shown in the following table:

| Sample | Total Reads | Clean Reads | Clean Ratio(%) | no rRNA | rRNA Ratio(%) | no rRNA pair |
| --- | --- | --- | --- | --- | --- | --- |
| N1 | 61040110 | 60261454 | 0.987243535 | 57415878 | 0.0472205 | 57415878 |
| N2 | 66716308 | 65835924 | 0.986804066 | 63789870 | 0.031078078 | 63789870 |
| N3 | 73608060 | 72636880 | 0.986806064 | 68607790 | 0.05546893 | 68607790 |
| N4 | 95125742 | 94663244 | 0.995138035 | 68785960 | 0.273361475 | 68785960 |
| N5 | 134408810 | 133154170 | 0.990665493 | 90640908 | 0.319278487 | 90640908 |
| L1 | 67751576 | 66558642 | 0.982392528 | 63751778 | 0.042171293 | 63751778 |
| L2 | 74670726 | 73582648 | 0.985428319 | 71502450 | 0.028270225 | 71502450 |
| L3 | 65000698 | 64045268 | 0.985301235 | 62630446 | 0.022090969 | 62630446 |
| L4 | 151466750 | 149728260 | 0.988522299 | 105847774 | 0.293067494 | 105847774 |
| L5 | 128764120 | 127498338 | 0.990169762 | 79016472 | 0.380254886 | 79016472 |

2. About Mapping: After obtaining clean reads, they need to be aligned to the known reference genome to determine what gene each read represents and its specific location on the genome. This process is called sequence alignment or mapping.

| sample | total_reads | mapped_reads | pair_mapped_reads | single_mapped_reads | mapped_ratio |
| --- | --- | --- | --- | --- | --- |
| N1 | 57415878 | 53872623 | 52862890 | 1009733 | 0.93828789 |
| N2 | 63789870 | 60084365 | 58945304 | 1139061 | 0.941910761 |
| N3 | 68607790 | 63762589 | 62424444 | 1338145 | 0.929378267 |
| N4 | 68785960 | 59547072 | 58645418 | 901654 | 0.865686428 |
| N5 | 90640908 | 79564364 | 78278518 | 1285846 | 0.877797517 |
| L1 | 63751778 | 59461696 | 58081758 | 1379938 | 0.932706473 |
| L2 | 71502450 | 63018785 | 61859456 | 1159329 | 0.881351408 |
| L3 | 62630446 | 58133700 | 56931426 | 1202274 | 0.928201916 |
| L4 | 105847774 | 95656671 | 93496728 | 2159943 | 0.903719251 |
| L5 | 79016472 | 71047870 | 69935056 | 1112814 | 0.899152648 |
